# Acoustic window planning for ultrasound acquisition

**DOI:** 10.1007/s11548-017-1551-3

**Published:** 2017-03-11

**Authors:** Rüdiger Göbl, Salvatore Virga, Julia Rackerseder, Benjamin Frisch, Nassir Navab, Christoph Hennersperger

**Affiliations:** 1 Computer Aided Medical Procedures, Technische Universität München, Boltzmannstr. 3, 85748 Garching, Germany; 2 Johns Hopkins University, 3400 North Charles Street, Baltimore, MD 21218 USA

**Keywords:** Planning framework, Automatic acquisitions, Robotic imaging, Ultrasonic imaging, Sonography

## Abstract

**Abstract:**

Autonomous robotic ultrasound has recently gained considerable interest, especially for collaborative applications. Existing methods for acquisition trajectory planning are solely based on geometrical considerations, such as the pose of the transducer with respect to the patient surface.

**Purpose:**

This work aims at establishing acoustic window planning to enable autonomous ultrasound acquisitions of anatomies with restricted acoustic windows, such as the liver or the heart.

**Methods:**

We propose a fully automatic approach for the planning of acquisition trajectories, which only requires information about the target region as well as existing tomographic imaging data, such as X-ray computed tomography. The framework integrates both geometrical and physics-based constraints to estimate the best ultrasound acquisition trajectories with respect to the available acoustic windows. We evaluate the developed method using virtual planning scenarios based on real patient data as well as for real robotic ultrasound acquisitions on a tissue-mimicking phantom.

**Results:**

The proposed method yields superior image quality in comparison with a naive planning approach, while maintaining the necessary coverage of the target.

**Conclusion:**

We demonstrate that by taking image formation properties into account acquisition planning methods can outperform naive plannings. Furthermore, we show the need for such planning techniques, since naive approaches are not sufficient as they do not take the expected image quality into account.

## Introduction

Sonography is a fundamental imaging modality for chronic cancerous [11] and non-cancerous [14] liver diseases. Novel developments in ultrasound (US) research, such as perfusion imaging [1], further contribute to its importance as a screening and interventional imaging device. Its main drawback, high operator variability, could be overcome by a robotic US imaging approach, that would allow for reproducible and precise data acquisition [6]. This will enable improved longitudinal studies and automated interventional US imaging, providing versatilities similar to other interventional imaging modalities such as cone-beam CT (CBCT), frequently employed in clinical practice.

**Fig. 1:**
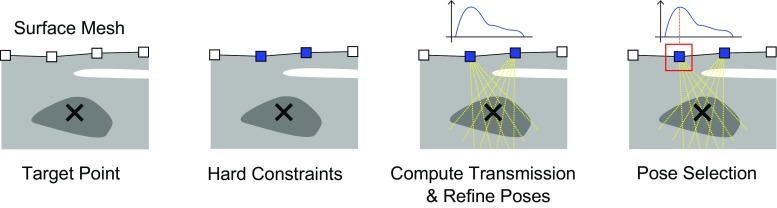
Schematic workflow of our method. For a defined target point, pose candidates are selected according to the hard constraints given by the US probe and acquisition properties. Next, the transmission of acoustic waves is optimized such that the best possible pose is selected to cover a defined target point

Aiming at widespread applications, however, the automatic planning of US trajectories needs to be addressed not only in 2D, but also in 3D. The latter provides crucial information in a number of clinical settings, such as diagnosis of hepatic diseases [3]. For planning, both the US probe position and its orientation heavily impact the resulting image quality. Consequently, one has to account for the directional physics of US imaging with acoustic attenuation, potential shadowing, and other imaging artifacts such as reverberations. Beyond the basic US-related constraints, the probe position planning needs to consider anatomical constraints such as the patient surface, and optimize for the resulting (expected) image quality to avoid adverse objects (e.g., bones in US). Only this way, an optimal acquisition can be performed for a given target anatomy. While a generalized planning can still be considered for easily accessible organs and structures such as the carotid artery, here we focus specifically on automatic acquisitions for organs with non-trivial acoustic windows, such as the liver or the heart.

Despite first approaches to US acquisition trajectory planning [4], a full optimization of 2D- and 3D-US acquisition trajectories with respect to the resulting image quality was to our knowledge not considered so far. In this regard, we introduce a novel planning framework for autonomous 2D- and 3D-US, and include geometrical, anatomy-based, and imaging-physics-based constraints to automatically retrieve the optimal position and orientation for a specific target point of interest. To optimize for the image quality, we integrate US attenuation estimates in our planning, which are derived from existing tomographic data such as CT and MRI. Ultimately, we aim at closing the gap for US trajectory planning, making autonomous US imaging more versatile.

### Related work

In view of prior work covering planning of automatic US acquisitions, a general US probe path planning is proposed in [4]. The method allows for the full coverage of a region of interest, but does not consider the resulting image quality of the planned acquisition to optimize for appropriate acoustic windows. More recently, [6] and [15] showed the feasibility and accuracy of autonomous US acquisitions performed by a robotic system, introducing concepts for constant force acquisitions with lightweight robots. The focus of these studies was, however, not on planning of the US trajectories based on the optimization of acoustic windows, but on their actual execution. The proposed systems employ US confidence maps [7–9] to estimate the quality of acquired US images, which can only be used during the acquisition itself but not for quality simulation. Thus, they cannot provide a global optimal planning. For a targeted quality optimization in a planning stage, US simulation approaches as in [10, 12, 16] make use of the physics-based properties of US imaging and focus on the synthesis of realistic images or images with realistic appearance. They do not provide a measure of image quality to assess the anatomical constraints linked to determining the best acoustic window for an acquisition.

## Acoustic window planning

In this section we introduce a method to determine optimized US probe positions for the acquisition of single US images. We show how a US sensor model can be used to integrate hard constraints, allowing the automatic planning of acquisitions for a target point (or structure) with a predefined US probe (“Sensor model” section). On this basis, we describe how acoustic transmission estimates can be used to retrieve the best acoustic window for a target structure (“Attenuation estimation” section). Finally, the overall probe position planning is described, which incorporates the aforementioned parameters for optimization of the probe position and its orientation (“Probe position planning” section). An overview of the proposed planning workflow is depicted in Fig. 1.

### Sensor model

US imaging imposes certain requirements with respect to the probe positioning based on the underlying US imaging physics (longitudinal acoustic waves), the acquisition parameters (wavelength, field of view) as well as the transducers hardware design (piezo element size and array shape). During their training, physicians learn to intuitively regard for all these parameters to identify reasonable acoustic windows for certain target structures. To mimic a similar behavior for automatized acquisition planning, we model a set of constraints for US pose optimization and evaluate a set of pose candidates with respect to the expected image quality. While 3D-US allows for improved structural coverage by acquiring volumetric information, at first we specifically focus on the foundation of 2D US imaging, and later extend the concept to 3D-imaging with freehand sweeps in (“Planning of 3D-trajectories” section)

**Fig. 2:**
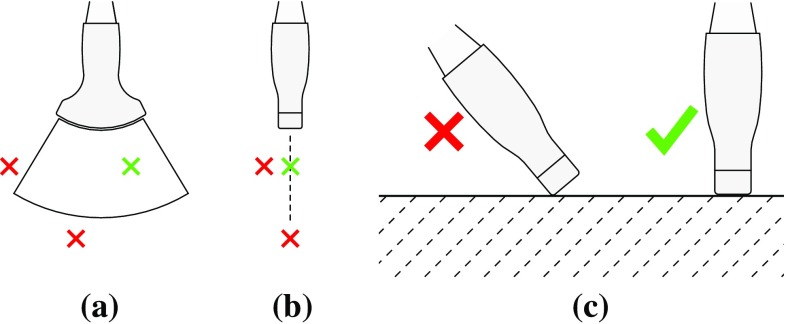
Constraints for US probe placement, as determined by the image plane in axiolateral **(a)** and elevational **(b)** directions, as well as by the need for correct acoustic coupling of the US probe **(c)**

The task of single-view probe position planning is to find a corresponding US probe pose $T_{\text {US}}$ to allow for *optimal* imaging of a target point $P_t$ defined by the operator. For automatic acquisitions, we assume that preoperative images are employed for the planning, in our case X-ray computed tomography (CT). From these images, a target point or structure is selected, and the probe positioning planned accordingly. For US imaging, resulting images are defined by the system settings (e.g., penetration depth $d_y$, frequency) and the probe geometry. With the discrete patient surface ${\mathcal {S}}$ extracted from the CT, we select a subset ${\mathcal {S}}_C$ of points $s \in {\mathcal {S}}$ for which the target point can be imaged based on these parameters. This is influenced by the image depth and the axial orientation of the probe with respect to the corresponding surface normals $n_s$

1 $$\begin{aligned} {\mathcal {S}}_C = \left\{ s \in {\mathcal {S}}:\, |P_t - s|< d_y\, \wedge \, \left\langle d_a, n_s \right\rangle < \alpha \right\} , \end{aligned}$$

with the transducers axial orientation $d_a$ restricted to the connection to the target point $(P_t - s)/|P_t - s|$. By doing this, one ensures that the target is always within the image plane, given the depth-constraint is fulfilled.

Figure 2 shows intuitively how these constraints are enforced in order to allow for US probe planning. To maintain sufficient acoustic coupling and ensure patient comfort, we restrict the angle between the target (patient) surface and the US probe. This limit depends on a number of factors including the transducers physical geometry and the stiffness of the covering tissue-layers. During our experiments we used $\alpha = \mathrm{cos}(30\,^\circ )$. These constraints thus guarantee that the region of interest is inside the image and that the insonificating pulse can reach the patients skin.

### Attenuation estimation

The identified pose candidates allow for the acquisition of images for a target region of interest, yet they do not regard for features of the patient anatomy, which could heavily influence the US image quality. In order to identify a suitable acoustic window for a target point, one has to ensure that sound waves reach the target with sufficient wave intensity. This thus corresponds to finding a linear path to the target that exhibits low attenuation while traversing the tissue.

Building on the efforts of [16], we propose an attenuation estimation based on CT, which is employed for acoustic window planning to evaluate possible poses as identified in (“Sensor model” section) with respect to their expected image quality. The acoustic transmission coefficient of US waves through an interface of two tissues with acoustic impedances $Z_1$ and $Z_2$ can be written as

2 $$\begin{aligned} t(Z_1, Z_2) = 1 - \left( \frac{Z_2 - Z_1}{Z_2 + Z_1} \right) ^2. \end{aligned}$$

**Fig. 3:**
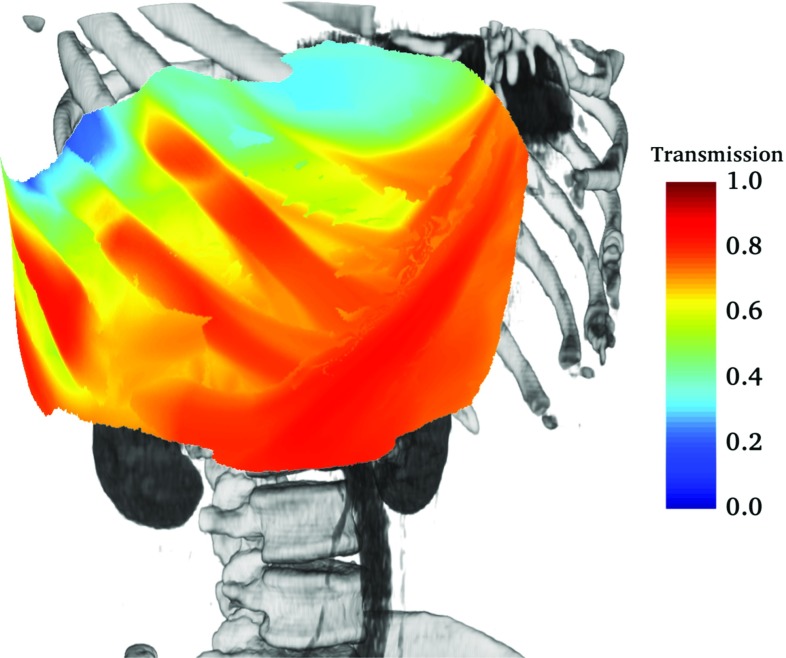
Mean transmission estimation ${\overline{t}}$ for one target point in the liver, drawn for each surface point

Using the approximately linear relationship between density $\rho$ and X-ray attenuation coefficient $\mu$ in tissues [13, 16], this can be rewritten as

3 $$\begin{aligned} {\Delta } t(x) = 1 - \left( \frac{ \left| {\Delta }\mu (x) \right| }{2 \mu (x)}\right) ^2, \end{aligned}$$

where a constant speed of sound is assumed for simplicity. US waves can traverse several tissue interfaces, such that the overall transmission from a base point *b* along a ray of direction *v* is

4 $$\begin{aligned} t(x) = \mathrm{exp} \left( -\int _0^a \left( \frac{ | {\Delta }\mu (b + l v) |}{2 \mu (b + l v)}\right) ^2 \mathrm {d} l \right) , \end{aligned}$$

with $x = b + av, a \in {\mathbb {R}}^+$. To account for the processing in common US imaging pipelines, a log-compression is applied to the transmission estimate

5 $$\begin{aligned} {\hat{t}}(x) = \frac{\mathrm{log}(1 + \nu t(x))}{\mathrm{log}(1 + \nu )}, \end{aligned}$$

with $\nu = 0.5$ representing a constant compression factor in this work, comparable to the findings in [16]. As the respective US probe geometry and resulting image geometries are known a-priori (number of elements $N_{el}$, scan-line origins w.r.t. the central element $b_i$ and their direction $v_i$), we can approximate the US transmission for a pose candidate and retrieve an average transmission value ${\overline{t}}$ for each relevant surface point $s \in {\mathcal {S}}_c$ and transducer orientation $R \in SO(3)$

6 $$\begin{aligned} {\overline{t}}(s, R) = \frac{1}{N_{el} d_y} \sum _{i = 1}^{N_{el}} \int \limits _0^{d_y} {\hat{t}}(R(b_i + l v_i)\, +s) \mathrm {d} l, \end{aligned}$$

thus computing the mean along all scan-lines. As the average transmission is mainly influenced by strong reflectors (e.g., bone) between the target structure and the respective surface point, this allows for the identification of surface points with higher wave intensities (and thus better signal to noise ratio) at the target structure. Figure 3 shows the exemplary mean transmission values estimated for each point on the surface for a target point inside the liver.

### Probe position planning

On the foundation of the hard constraints and the transmission model, we can retrieve a patient-specific optimal US pose to cover a target point with maximized acoustic intensity. Anatomical factors such as the ribs will cause strong reflections (i.e., low transmission) for certain probe orientations, whereas a change in its position potentially has smaller effects on the expected transmission. We propose a two-stage quality maximization, where the best angle is retrieved first, since the transmission values should be almost convex w.r.t. the probe angle. The surface position *s* is then optimized to retrieve the final US probe pose $T_{US} = ({\hat{R}}, {\hat{s}})$.

In our case, the US probe pose is already characterized by a base point on the patient surface $s_i \in {\mathcal {S}}$ and the axial orientation $d_a$. Therefore, the goal of the first optimization stage is to find the best transducer orientation $R_{\text {US}}$ for the acquisition in one degree of freedom. By defining the axial direction to be $d_a = (P_t - s_i) / |P_t - s_i|$, we effectively align the transducer center toward the target point and reduce the space of eligible orientations to the rotations around $d_a$. The mean transmission ${\overline{t}}$ can then be employed as quality metric in order to maximize the overall transmission for a given target position

7 $$\begin{aligned} (s, \phi ) = {\mathop {{{\mathrm{arg\,max}}}}\limits _{{(s, \phi ) \in {\mathcal {S}}_C \times [0,\ldots ,\pi [}}} {\overline{t}}(s, R_{d_\text {a}}(\phi )), \end{aligned}$$

where $R_{d_\text {a}}(\phi )$ is the rotation around the fixed axis $d_a$ by the angle $\phi$. Intuitively, by maximizing the transmission across one image and different image candidates, those with a low transmission are rated lower and discarded in the optimization.

**Fig. 4:**
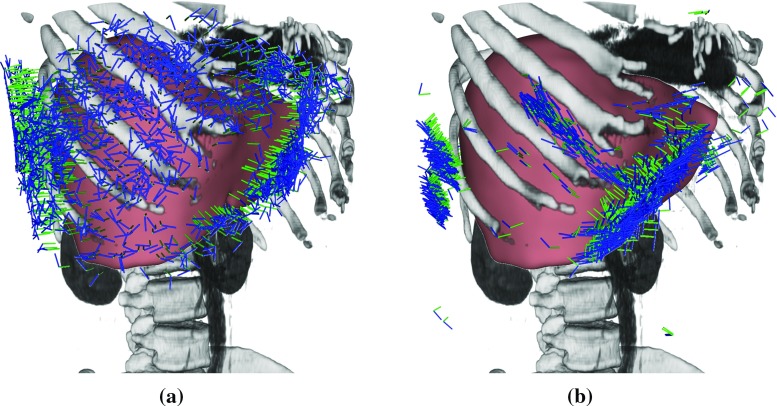
Comparison of single-view poses for the $N_s$ target points for one subject from the SLIVER07 dataset [5] together with a render of the underlying CT volume. The orientations of the image axes are visualized with *colored lines*, *green* for axial and *blue* for lateral directions. **a** shows the plans created naively. The results of our method are shown in **(b)**

### Planning of 3D-trajectories

Generalizing the proposed method for 3D-trajectories, we optimize the best acoustic window for the acquisition of all the $N_t$ target points $P_{ti}$ within a structure. The 3D-trajectories planned by our method are restricted to one base point $s \in S$, and only vary with respect to their orientation. By choosing a point that allows for high transmissions to all target points, we ensure that all poses are within an acoustic window.

8 $$\begin{aligned}&(s, (\phi _i,\ldots ,\phi _{N_t}))\nonumber \\&\quad = {\mathop {{{\mathrm{arg\,max}}}}\limits _{{(s, (\phi _i,\ldots ,\phi _{N_t})) \in {\mathcal {S}}_{T} \times ([0, \ldots , \pi [)^{N_t}}}} \prod _{i=1}^{N_t} {\overline{t}}(s, R_{d_\text {a}}(\phi _i)), \end{aligned}$$

where the set of surface points has to fulfill the hard constraints w.r.t. every target point ${\mathcal {S}}_{T} = \bigcap _{i=1}^{N_t} {\mathcal {S}}_{Ci}$. We follow the same two-step maximization approach as above by first selecting the best rotations for each target point and surface point $s \in {\mathcal {S}}_{T}$, followed by selecting the base point with the overall best transmission.

## Experiments

As acoustic window planning has not been considered so far, we compare the results of the proposed planning framework to a naive planning, comparable to the planning approaches in [4, 6]. This consists of choosing the surface point $s_n$ nearest to the target point $P_t$ as base point for the acquisition. The transducer orientation $d_a$ is then chosen in the same way as described in (“Probe position planning” section), while minimizing the angle between the transducer and the surface normal $n_n$ at $s_n$. By doing so, the transducers lateral axis $d_l$ is $(n_n \times d_a) / |n_n \times d_a|$. When multiple views are considered, the naive approach is to choose the base point and $d_a$ as for single-views and the elevational direction $d_e$ as the rejection of the input-trajectory direction $d_T$ from $d_a$, aiming at trajectories with parallel image planes.

9 $$\begin{aligned} d_e = \frac{d_T - d_a \left\langle d_a, d_T\right\rangle }{|d_T - d_a \left\langle d_a, d_T \right\rangle |} \end{aligned}$$

We first perform a set of experiments on publicly available datasets (“Synthetic planning” section), as well as for a torso (rib) phantom, where scans are performed with a robotic US system (“Robotic acquisition experiments” section).

The planning was performed on a workstation (Intel i7-4820K, NVIDIA Titan Black) and the computation of the mean transmission was implemented in CUDA. Computing the best poses for 10 target points took on average 356 seconds.

### Synthetic planning

Using a dataset of 20 annotated upper-torso CTs from the SLIVER07 challenge dataset [5] we performed an evaluation of the proposed single- and multi-view planning method. The volumes featured different portions of the thorax and abdomen respectively, but all contained the liver with some margin. For our evaluation, we chose $N_s = 2000$ random points inside the liver segmentations and manually defined between 4 and 6 plans for each volume to cover the large vessel trees. This resulted in a total of 100 multi-view plans for all evaluated datasets.

#### Stability

We demonstrate the effectiveness of our acoustic window planning approach qualitatively by independently computing the best poses for the $N_s$ random points of one case. Figure 4 shows the resulting pose in combination with a visualization of the corresponding CT volume. The poses computed with the naive approach are widely spread over the thorax and a significant number of image planes intersect with ribs, resulting in a majority of images with strong reflectors and prominent artifacts in images. The proposed method results in poses that closely follow the acoustic windows in the intercostal spaces. Inferior to the costal cartilage, the image planes are oriented tangent to the rib cage, suggesting that the planned poses are suited better for US imaging.

**Fig. 5:**
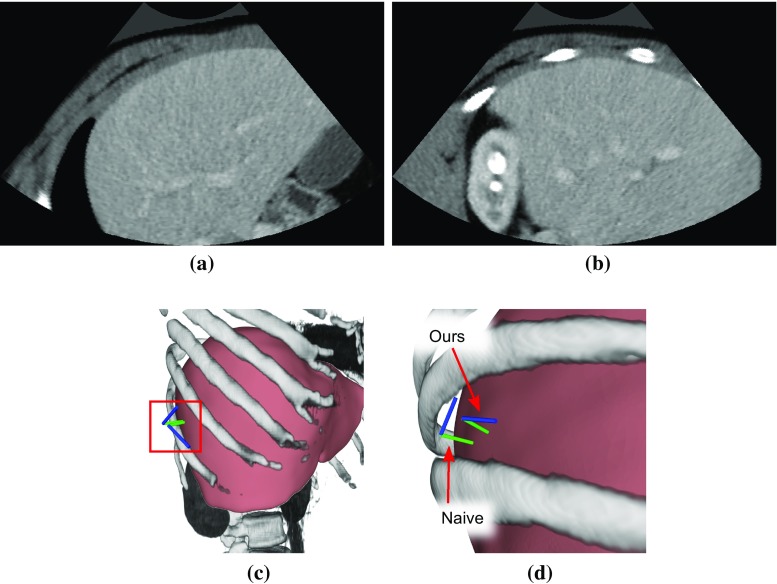
Comparison of synthetic poses for one target point. **a** Shows the CT slice at the proposed pose, **b** at the one resulting from naive planning. **c**, **d** Set the poses in relation to the surrounding anatomy

**Table 1:** Acoustic window quality mean and SD for the naive and our planning technique applied to single- and multi-view settings

	${\overline{r}}_{\text {ct}}$ Ours	${\overline{r}}_{\text {ct}}$ Naive	${\overline{r}}_{\text {seg}}$ Ours	${\overline{r}}_{\text {seg}}$ Naive
Single-view planning	$0.222 \pm 0.163$	$0.294 \pm 0.174$	$0.488 \pm 0.171$	$0.477 \pm 0.161$
Multi-view planning	$0.214 \pm 0.175$	$0.256 \pm 0.179$	$0.470 \pm 0.225$	$0.456 \pm 0.214$

Columns **1** and **2** Show the ratios of non-soft-tissue values, columns **3** and **4** The ratios of depicted liver

#### Quality of acoustic window

A quantitative comparison of both planning methods was performed using the ratio of non-soft-tissue areas $r_{\text {CT}}$ and the ratio of depicted liver $r_{\text {seg}}$, as given by the segmentation,

10 $$\begin{aligned} r_{\text {CT}}&= \tfrac{1}{n} \left| \left\{ x:\, \mu (x) < \beta _1\, \vee \mu (x) > \beta _2 \right\} \right| \end{aligned}$$

11 $$\begin{aligned} r_{\text {seg}}&= \tfrac{1}{n} \left| \left\{ x:\, x \in \text {segmentation} \right\} \right| . \end{aligned}$$

Where *n* is the number of pixels in the image, and $\beta _1 = -100\,\text {HU}, \beta _2 = 150\,\text {HU}$ separating soft from hard tissue following [13]. The ratio of non-soft-tissue $r_{\text {CT}}$ indicates the fraction of dense tissues (i.e., bones or air-filled areas) contained in the image, which impairs the transmission of US pulses across soft-tissues. Consequently, a lower ratio exhibits potentially better overall image quality, as a large $r_{\text {CT}}$ potentially also causes US artifacts. Conversely, the ratio of the target organ $r_{\text {seg}}$ depicted in a target image serves as measure of how much anatomical context is provided in the resulting US images. A higher $r_{\text {seg}}$ provides more context, as a higher fraction of the target organ is covered by the image content.

The evaluation was performed on CT slices at the respective pose with the size of the US image, as shown in Fig. 5. We used the single- and multi-view plans created using the segmentations of each of the 20 datasets. Table 1 shows the quantitative results for both evaluated measures. As the mean ratio of non-soft-tissues is lower for our method, while the ratios of depicted target anatomy are roughly equal, this effectively shows that the poses resulting from our method are less likely to have shadowing artifacts, while allowing for a similar coverage of the target image region. In Fig. 5, the pose obtained with naive planning cuts through several ribs, which would result in strong shadows in an US image. Our method planned a pose that only contains non-optimal regions at the bottom and is likely to have no shadowing artifacts in the liver.

### Robotic acquisition experiments

The two methods were evaluated on real acquisitions performed by a robotic US system. Intuitively, the manual execution of planned trajectories is prone to errors and would lack accuracy even in the case of statically placed phantoms. Furthermore, the reproducibility of such trajectories would be limited significantly. Robotic US well tackles these limitations and allows for the autonomous execution of planned probe trajectories. In this view, the robotic system previously presented in [6] and [15] represents a good choice to qualitatively and quantitatively assess the performance of our proposed planning technique for US acquisitions.

**Fig. 6:**
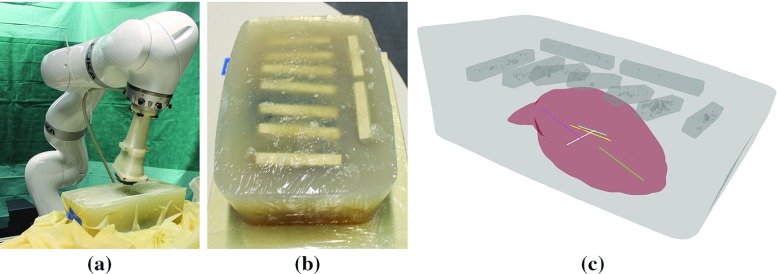
Phantom and robotic system used for experiments. **a** Robotic arm equipped with US transducer approaching the phantom covered by a thin latex sheet. **b** Gelatin-agar phantom, chalk bars resembling ribs are visible. **c** Rendering of the phantom, the selected trajectories are shown in *distinct* colors

**Fig. 7:**
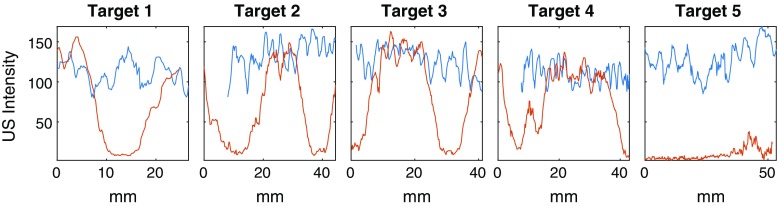
US intensities along planned trajectories in phantom for naive and the proposed planning technique, ours in *blue*, naive in *orange*. For target points that were not covered by the acquisition, no value is shown

The system is composed of a robotic arm, KUKA LBR iiwa R800 (KUKA Roboter GmbH, Augsburg, Germany), controlled using a custom software module^1^ and the robot operating system (ROS) framework. An RGB-D sensor (Kinect, Microsoft Corporation, Redmond, WA, USA) is positioned above the examination bed and used to perform CT to phantom calibration. The US acquisitions are obtained from an Ultrasonix$^\circledR$ Sonix RP US system equipped with a 4DC7-3/40 curvilinear transducer, using the following parameters: frequency: 3.3 MHz, depth: 140 mm, gain: 50%. ROS and the respective modules to control the robot ran on a PC (Intel Core i5, NVIDIA GTX 970) communicating with the planning system.

Using this system, the acoustic window planning was evaluated for single- and multi-view acquisitions on a gelatin–agar phantom based on [2]. The tissue-mimicking material is targeted to multimodal imaging. A liver was molded from a gel made out of 10 weight percent (wt%) gelatin and 6 wt% agar, diluted in water. For enhanced scattering in US 0.6 wt% graphite powder was added. The surrounding tissue-mimicking gel consisted of 3 and 1.5 wt% gelatin and agar, respectively. Shortly before complete solidification of the gel, the liver model and chalk sticks to simulate ribs were added.

Acquisitions were planned in a CT volume of the phantom, and target acquisition trajectories were computed both with our and the naive planning method. The five target lines (27.0–53.5 mm) defined for the acquisitions with differing orientations w.r.t. the ribs are shown in Fig. 6c. After planning, the trajectories were executed by the robotic setup as in [6]. A constant force of 5 N onto the phantom’s surface was applied for all US acquisitions. They were reconstructed into 3D volumes (compounding) and employed for evaluation.

Figure 7 shows the US intensity profiles measured along the five defined target lines. Blue plots display the intensities from acquisitions planned with our method, while the orange plots show the naively planned ones. Where target points were not covered by the US acquisition, no values are shown. Such regions result from inherent inaccuracies of the surface to surface registration employed in [6], as well as by tissue deformation. It can be clearly seen that naively planned volumes exhibit high variations of intensity along the line within homogeneous tissue, while ours show a continuously high visibility, only containing speckle variations.

**Fig. 8:**
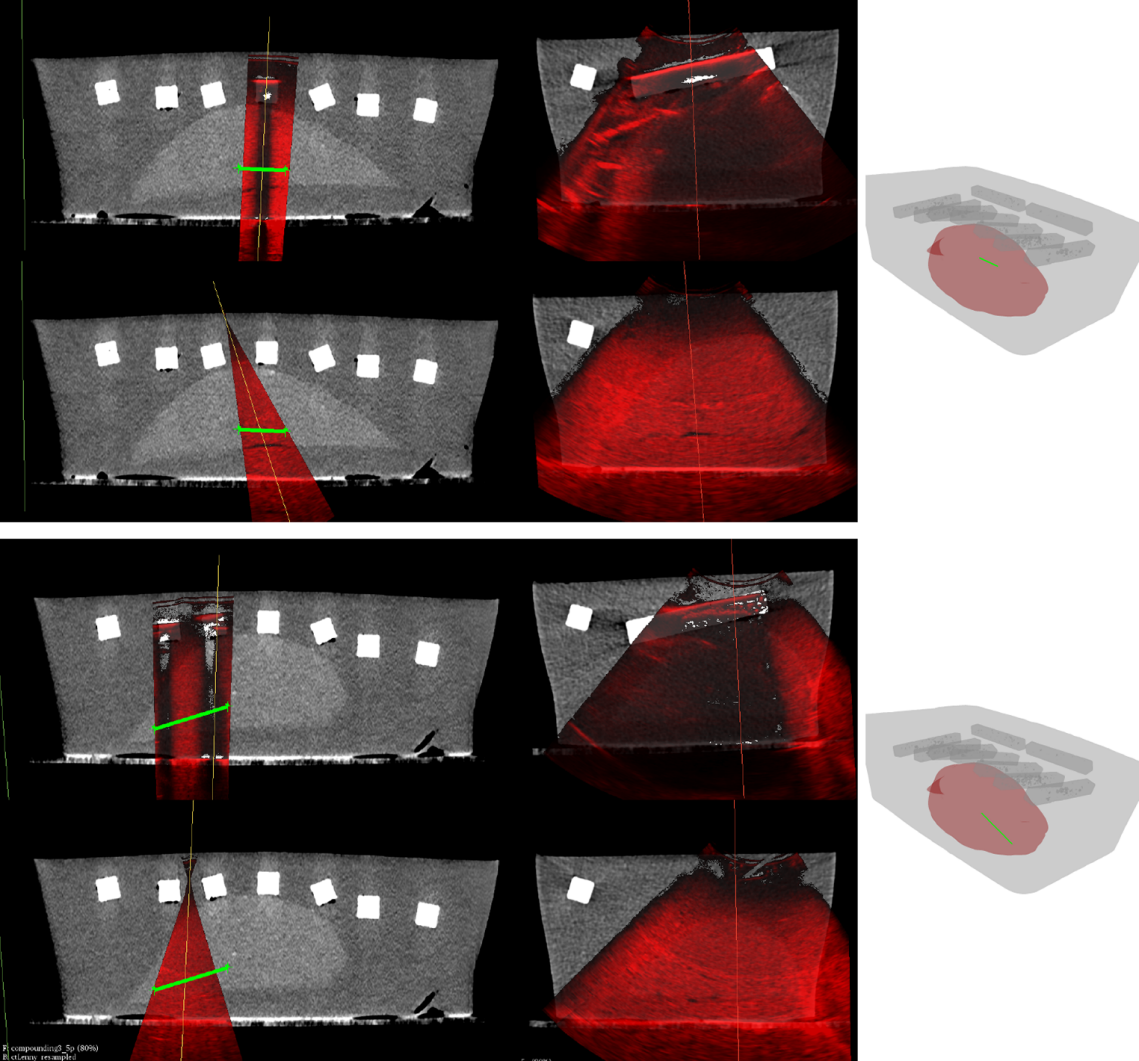
Phantom US acquisitions (*red*) for two trajectories using the CT for planning. Rows 1 and 3 show the naive sweeps, rows 2 and 4 sweep according to our planning, each in different planes (*first* and *second* column). The positioning of the target points is shown as *green line* in the rendering and the slices

Slices of two acquisitions are shown in Fig. 8 together with the target lines inside of the volume, comparing naive planning to our method. As it can be observed, the naively planned trajectories cross bones and the resulting images are subject to strong shadowing artifacts in these locations. In contrast to this, the plans created with our method allow for imaging of the selected structures without shadowing throughout the sweep.

## Discussion

On the foundation of our analysis and presented work, the results of both the synthetic trajectory planning as well as the phantom acquisitions show the need for trajectory planning approaches, considering the inner anatomy for optimizing the image quality. Based on the physics of the US image formation process, the introduced maximization of the expected image quality by acoustic transmission allows for the planning of single- and multi-view acquisitions not affected by shadowing artifacts, as we have shown. With this work, we aim at providing the basis for truly autonomous US acquisitions of a variety of anatomies that could not be imaged previously due to restricted acoustic windows. Beyond that, the proposed method could also be used to train US technicians, providing trainees with feedback on the transducer positioning.

While our method provides optimal acquisition plans, their execution requires an accurate patient registration, as acoustic windows can be of limited size. For the example of cardiac US, a planning in the intercostal space needs to be precise w.r.t. the patient registration, as otherwise images would be distorted significantly by the ribs. This becomes even more important, as respiratory motion could potentially require a continuous update of the patient registration in view of imaging in-vivo.

With this basis, our future work will include a more precise patient registration, possibly adding image based registration refinement as proposed in [6] and incorporating methods to detect degraded image quality during the acquisition [8]. This would allow the system to exclude affected images from the 3D compounding as well as the extension of our method for the planning of longer US trajectories with varying base points. Excluding degraded images based on their actual quality can reduce the impact of changes which inherently could not be accounted for in the planning, such as the presence of bowel gas or lesions resulting from the current treatment. Those changes could cause significant artifacts and attenuation.

The observed computation times do not allow for an interactive execution, but since the trajectories are meant to be obtained preoperatively, this is not critical. Nevertheless, we plan to reduce the computational cost in the future.

Finally, our future work includes the generalization of the proposed method to use other tomographic modalities such as MRI as basis for the transmission estimation [12] and ultimately develop it toward an atlas-based approach as in [15]. We also aim at integrating further factors into the optimization, such as the volumetric coverage and anatomical context provided by the trajectory. To this end, a proof-of-concept study involving human acquisitions will be necessary to show the validity of the approach in a clinical setting, where CT data are a prerequisite for a clinical trial.

## Conclusion

In this work, we presented the first fully automatic trajectory planning approach for autonomous and collaborative robotic US acquisitions, which takes the expected image quality into account. We have demonstrated both the theoretical and practical advantages of the proposed approach over conventional planning techniques. The method was evaluated using 20 virtual planning scenarios based on real patient data as well as five real acquisitions scenarios on a realistic tissue-mimicking phantom. In particular, we demonstrated that the proposed method achieves a higher acoustic window quality throughout the acquired sweeps in comparison with a naive planning approach, while yielding a comparable coverage of the target anatomy. Conducted phantom experiments further showed that this advantage can also be observed for robotic US imaging, as indicated by more constant intensity signals along the planned trajectory.

## Footnotes

1 https://github.com/SalvoVirga/iiwa_stack.

## References

[1] Christensen-Jeffries K, Browning RJ, Tang MX, Dunsby C, Eckersley RJ (2015). In vivo acoustic super-resolution and super-resolved velocity mapping using microbubbles. IEEE Trans Med Imaging.

[2] Dang J, Frisch B, Lasaygues P, Zhang D, Tavernier S, Felix N, Lecoq P, Auffray E, Varela J, Mensah S, Wan M (2011) Development of an anthropomorphic breast phantom for combined PET, b-mode ultrasound and elastographic imaging. IEEE Trans Nucl Sci 58(3):660–667

[3] Esmat G (2000) Three-dimensional ultrasonography in hepatogastroenterology. In: Kurjak A, Kupesic S (eds) Clinical Application of 3D Sonography. CRC Press, ISBN: 9781842140062

[4] Graumann C, Fuerst B, Hennersperger C, Bork F, Navab N (2016) Robotic ultrasound trajectory planning for volume of interest coverage. In: 2016 IEEE international conference on IEEE Robotics and automation (ICRA), pp 736–741

[5] Heimann T, Van Ginneken B, Styner MA, Arzhaeva Y, Aurich V, Bauer C, Beck A, Becker C, Beichel R, Bekes G, Bello F, Binnig G, Bischof H, Bornik A, Cashman PMM, Chi Y, Córdova A, Dawant BM, Fidrich M, Furst JD, Furukawa D, Grenacher L, Hornegger J, Kainmüller D, Kitney RI, Kobatake H, Lamecker H, Lange T, Lee J, Lennon B, Li R, Li S, Meinzer HP, Németh G, Raicu DS, Rau AM, Van Rikxoort EM, Rousson M, Ruskó L, Saddi KA, Schmidt G, Seghers D, Shimizu A, Slagmolen P, Sorantin E, Soza G, Susomboon R, Waite JM, Wimmer A, Wolf I (2009). Comparison and evaluation of methods for liver segmentation from CT datasets. IEEE Trans Med Imaging.

[6] Hennersperger C, Fuerst B, Virga S, Zettinig O, Frisch B, Neff T, Navab N (2016) Towards MRI-based autonomous robotic US acquisitions: a first feasibility study. IEEE Trans Med Imaging 36(2):538–548. doi:10.1109/tmi.2016.2620723 10.1109/TMI.2016.2620723 27831861

[7] Hennersperger C, Mateus D, Baust M, Navab N (2014) A quadratic energy minimization framework for signal loss estimation from arbitrarily sampled ultrasound data. In: International conference on medical image computing and computer-assisted intervention. Springer, Berlin, pp 373–380 10.1007/978-3-319-10470-6_47 25485401

[8] Karamalis A, Wein W, Klein T, Navab N (2012). Ultrasound confidence maps using random walks. Med Image Anal.

[9] Klein T, Wells III WM (2015) RF ultrasound distribution-based confidence maps. In: International conference on medical image computing and computer-assisted intervention. Springer International Publishing, Berlin, pp 595–602

[10] Kraft S, Conjeti S, Noël PB, Carlier S, Navab N, Katouzian A (2014) Full-wave intravascular ultrasound simulation from histology. In: International conference on medical image computing and computer-assisted intervention. Springer, Berlin, pp 627–634 10.1007/978-3-319-10470-6_78 25485432

[11] Makuuchi M, Hasegawa H, Yamazaki S, Takayasu K, Moriyama N (1987). The use of operative ultrasound as an aid to liver resection in patients with hepatocellular carcinoma. World J Surg.

[12] Salehi M, Ahmadi SA, Prevost R, Navab N, Wein W (2015) Patient-specific 3D ultrasound simulation based on convolutional ray-tracing and appearance optimization. In: International conference on medical image computing and computer-assisted intervention. Springer, Berlin, pp 510–518

[13] Schneider U, Pedroni E, Lomax A (1996). The calibration of CT hounsfield units for radiotherapy treatment planning. Phys Med Biol.

[14] Tchelepi H, Ralls PW, Radin R, Grant E (2002). Sonography of diffuse liver disease. J Ultrasound Med.

[15] Virga S, Zettinig O, Esposito M, Pfister K, Frisch B, Neff T, Navab N, Hennersperger C (2016) Automatic force-compliant robotic ultrasound screening of abdominal aortic aneurysms. In: Proceedings IROS 2016 IEEE international conference on intelligent robots and systems

[16] Wein W, Brunke S, Khamene A, Callstrom MR, Navab N (2008). Automatic CT-ultrasound registration for diagnostic imaging and image-guided intervention. Medi Image Anal.

